# T-Score and Handgrip Strength Association for the Diagnosis of Osteosarcopenia: A Systematic Review and Meta-Analysis

**DOI:** 10.3390/jcm10122597

**Published:** 2021-06-12

**Authors:** Umberto Tarantino, Chiara Greggi, Virginia Veronica Visconti, Ida Cariati, Mariagrazia Tallarico, Matteo Fauceglia, Riccardo Iundusi, Marco Albanese, Carlo Chiaramonte, Elena Gasbarra

**Affiliations:** 1 Department of Clinical Sciences and Translational Medicine, “Tor Vergata” University of Rome, Via Montpellier 1, 00133 Rome, Italy; chiara.greggi@gmail.com (C.G.); ida.cariati@uniroma2.it (I.C.); 2 Department of Orthopaedics and Traumatology, “Policlinico Tor Vergata” Foundation, Viale Oxford 81, 00133 Rome, Italy; virginia.veronica.visconti@uniroma2.it (V.V.V.); mariagraziatallarico@gmail.com (M.T.); matfauc@gmail.com (M.F.); riccardo.iundusi@uniroma2.it (R.I.); gasbarra@med.uniroma2.it (E.G.); 3 Department of Biomedicine and Prevention, Medical Genetics Section, University of Rome “Tor Vergata”, 00133 Rome, Italy; 4 Department of Statistics, University of Rome “Tor Vergata”, 00133 Rome, Italy; marco.albanese@uniroma2.eu (M.A.); chiaramonte.carlo43@gmail.com (C.C.)

**Keywords:** systematic review, meta-analysis, osteosarcopenia, sarcopenia, osteoporosis, T-score, handgrip strength

## Abstract

Background: Osteosarcopenia is a recently identified condition caused by the coexistence of osteoporosis and sarcopenia that affects the frail elderly population, leading to an increased risk of falls and fractures. Given the recent socio-economic interest associated with osteosarcopenia, the aim of this meta-analysis is to provide an overview of the factors potentially involved in its pathogenesis, assessing its population type, prevalence, and associated variables. Methods: A comprehensive systematic search for relevant studies, published from 2015 to 2020, was performed by using PubMed, EMBASE, and Cochrane databases. We analysed the variables of age, vitamin D, handgrip, and T-score in four different groups: healthy, osteopenic–osteoporotic, sarcopenic, and osteosarcopenic. Results: A total of 6504 patients from 16 studies were included in the final meta-analysis. The analysis of the individual variables reveals a statistically significant correlation between the handgrip test data and T-score (*p* < 0.001). Conclusions: The correlation between T-score values and handgrip strength suggests a new potential parameter in the development of predictive models that could be used in clinical practice, highlighting its importance for the diagnosis of osteosarcopenia.

## 1. Introduction

The concept of “osteosarcopenia”, which emerged in 2009 with the first editorial by Binkley and Buehring [1], is currently used to refer to a subgroup of elderly patients with osteoporosis and sarcopenia, characterised by an higher risk of falls, fractures, disability, and frailty [2]. Osteoporosis, defined by low bone mass and micro-architectural deterioration of bone tissue [3], and sarcopenia, manifested by loss of muscle mass, strength, and function [4], often coexist in the fragile population, leading to significantly worse outcomes than those observed in either condition alone. Several evidences demonstrate considerable overlap in the pathophysiology of osteoporosis and sarcopenia, suggesting the possibility of treating the conditions together [5,6,7,8]. Indeed, bone and muscle not only interact mechanically, but also communicate with each other biochemically, via complex paracrine and endocrine mechanisms, to maintain muscle and bone homeostasis [9,10,11]. Many scientists agree that the prevalence of osteosarcopenia increases with age: Fahimfar and colleagues reported that it occurs in 14.3% of men in the 60–64 age group and in 59.4% of men over 75; in women, the disease affects 20.3% of the 60–64 age group and 48.3% of women over 75 [12]. In addition, a higher prevalence of osteosarcopenia has been observed in women (25.5–82.6%) than in men (16.4–32.0%) [13]. Finally, in patients with low-energy trauma fractures, the prevalence of osteosarcopenia is between 17 and 96.3% in both sexes [12]. Sarcopenia is generally defined as a decrease in skeletal muscle mass and muscle strength or motor skills [11]. Several research groups have focused their attention on the study of this disease; among the most important are the EWGSOP2 (European Working Group on Sarcopenia in Older People 2) [14] of 2019 and the AWGS (Asian Working Group for Sarcopenia) [15] of 2014. The parameters used to make a diagnosis of sarcopenia are strength, muscle quantity, and physical performance [4]. Accurate measurement of grip strength (handgrip strength test) requires the use of a calibrated handheld dynamometer under well-defined test conditions with interpretative data from appropriate reference populations. Low grip strength is a powerful predictor of adverse patient outcomes, as well as long hospital stay, increased functional limitations, poor quality of life, and mortality. On the other hand, in relation to osteoporosis, the World Health Organisation (WHO) has developed diagnostic criteria using a score based on standard deviations of bone mineral density (BMD), referring to peak bone mass in young healthy women. On this basis, osteoporosis was defined by a BMD T-score of less than −2.5, and osteopenia by a T-score between −1 and −2.5. Undoubtedly, the diagnostic criteria have supported the importance of low BMD in the pathogenesis of fragility fractures and have provided a tool that can be used in epidemiological studies to quantify the prevalence of osteoporosis; however, the usefulness of BMD as a clinical indicator of osteoporosis is limited because it is only one of a number of risk factors related to possible fractures [16]. The focus on research and treatment of pathological conditions that can affect older people is certainly one of the health priorities for the near future. As this is a newly defined pathology, there are no currently valid diagnostic criteria that would allow the rapid elaboration of a diagnosis of osteosarcopenia. In this respect, our meta-analysis aims to analyse the correlation between some variables normally considered for the diagnosis of osteoporosis (T-score) and sarcopenia (handgrip strength test), with the final objective of defining one or more new parameters to refer to for the diagnosis of this pathology.

## 2. Methods

### 2.1. Search Strategy and Selection Criteria

**Figure 1:**
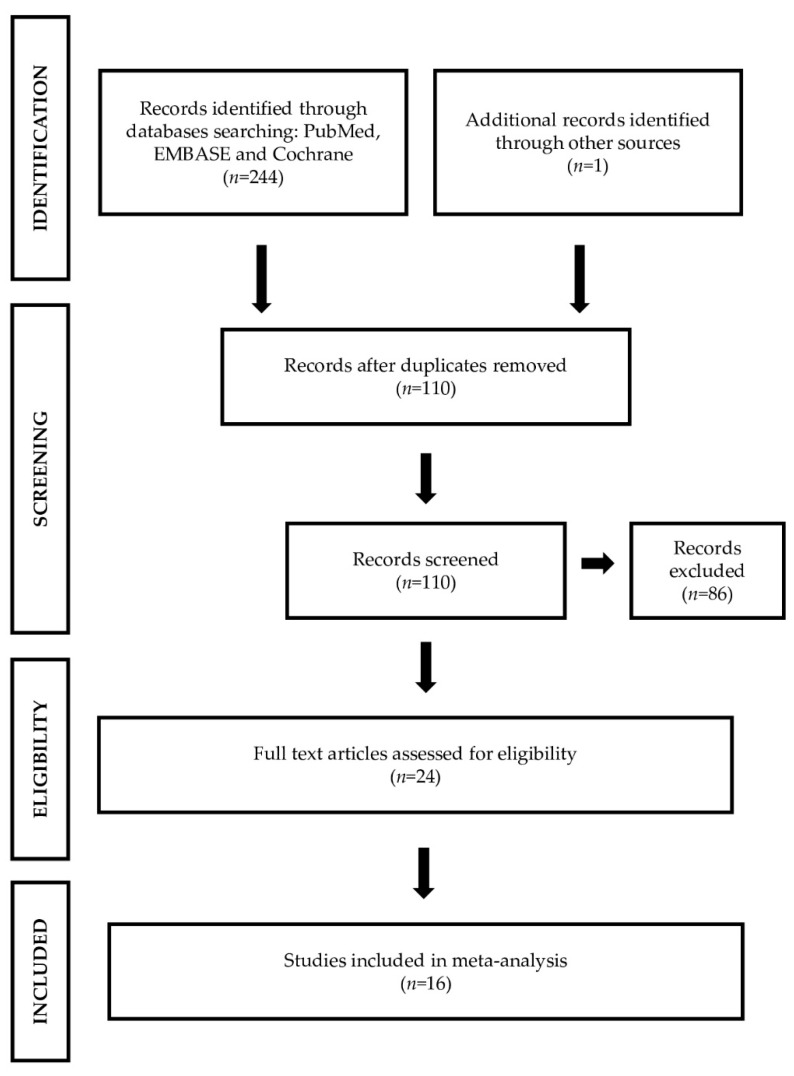
PRISMA flow diagram of the literature search and selection process of the included studies.

A systematic review was conducted following the recommendations of the Preferred Reporting Items for Systematic Reviews and Meta-Analyses (PRISMA) (Figure 1) [17]. An independent literature search was conducted across PubMed, EMBASE, and Cochrane databases. The search strategy was based on combination of following terms: “osteosarcopenia”, “sarco-osteopenia”, “osteopenia”, “osteoporosis”, “sarcopenia”, “fractures”, and “fragility”. Case reports, abstracts, short communications, letters to the editor, dissertations, and studies lacking case and control adequate numbers were also excluded. The main requirements for primary studies were introduced to have the most homogeneous data possible (same type of outcome, presence of clear, and explanatory summary tables as well as the use of statistical methodology), with the aim of searching for relationships between variables and risk groups. For selecting primary studies, we used criteria for classifying patients into clusters or homogeneous groups (Table 1).

### 2.2. Data Collection, Extraction, and Quality Assessment

Data were collected independently by two reviewers using a standardised data extraction form to summarise characteristics of the studies and outcome data [18]. Discrepancies or difficulties were resolved by consensus. From each individual study, we extracted baseline information: the first author’s name; the year of publication; socio-demographic data (type of population, sex ratio, mean age); sample size; study design; tools used to assess bone and muscle mass (handgrip strength test, gait speed test, timed up and go test); patient characteristics (BMI, BMD, T-score, serum levels of calcium, vitamin D, and PTH). To include as many studies as possible in our systematic review, we contacted authors or co-authors when information was missing in the full-text paper. After collection of the relevant articles, the Newcastle–Ottawa Scale (NOS) was used to assess their methodological quality.

**Table 1:** Characteristics of the studies.

References	Year	Study Design	Type of Participants	Mean/Median Age	Sample Size	*n*, Male	*n*, Female	Osteoporosis Diagnostic Criteria	Sarcopenia Diagnostic Criteria	*n*, Osteosarcopenic Subjects	%, Osteosarcopenic Subjects
[19]	2015	transversal	fragility, falls, fractures	NA	679	224	455	WHO	* *p* < 0.05	258	38%
[20]	2015	observational	hip fracture	82	239	72	167	WHO	AWGS	166	69%
[21]	2016	control cases	hip fracture	NA	359	87	272	WHO	AWGS	NA	NA
[22]	2016	prospective-observational	community living seniors	70.5	750	0	750	WHO	ALM < −DS	NA	NA
[23]	2016	observational	community living seniors	NA	68	21	47	WHO	EWGSOP	19	28%
[24]	2018	retrospective	hip fracture	77.8	324	78	246	WHO	AWGS	93	29%
[25]	2018	transversal	consecutively admitted to hospital, first hip fracture	81.4	80	80	0	WHO	* *p* < 0.05	NA	NA
[26]	2018	longitudinal	community living seniors	76.7	1575	1575	0	WHO	EWGSOP	131	8%
[27]	2019	transversal	hospitalised	80.6	141	57	84	WHO	EWGSOP	20	14%
[28]	2019	prospective-observational	community living seniors	78.8	106	34	72	WHO	* *p* < 0.05	NA	NA
[29]	2019	transversal	fragility, falls, fractures	77.9	253	57	196	WHO	EWGSOP2	138	55%
[30]	2019	prospective-transversal	postmenopausal subjects	64.14	140	0	140	WHO	EWGSOP	90	64%
[31]	2019	transversal	community living seniors	76	484	147	337	WHO	EWGSOP2	25	5%
[32]	2019	transversal	healthy subjects	71.4	427	205	222	* *p* < 0.05	* *p* < 0.05	36	8%
[13]	2020	transversal	community living healthy seniors	75	529	232	297	WHO	EWGSOP2	8	2%
[33]	2020	transversal	hip fracture	79.7	350	0	350	WHO	EWGSOP2	NA	NA

### 2.3. Data Analysis

The outcomes provided by the primary studies detected within each cluster were the numerosities and continuous measures such as means and standard deviations, both for clinical covariates (BMI, T-score, vitamin D, handgrip) and demographic covariates (age and gender) (Table 2). The weighted arithmetic averages of the outcomes of the primary studies were then calculated for each group with weights equal to the number of statistical units in the same study, named global outcome. If data were not available, the overall outcome values of the uncategorised group (RG) or the calculated overall outcome were assigned. Statistical analyses were conducted by using R software (R Foundation for Statistical Computing, version 3.6.2; 2019-12-12; Vienna, Austria). Pearson’s chi-squared test for evaluating the independence between covariates in the groups and *t*-test for Pearson correlation have been used. Differences were considered significant when the *p* value was <0.05 (* *p* < 0.05, ** *p* < 0.01, *** *p* < 0.001).

## 3. Results

### 3.1. Literature Search and Study Selection

The initial search yielded 245 studies, with duplicate studies removed, resulting in 110 studies remaining. Following the inclusion and exclusion criteria, we selected 16 studies. Figure 1 shows a flow diagram of the literature search and study selection.

### 3.2. Study Characteristics

The selected studies include different experimental designs: transversal, observational, control cases, prospective, retrospective, and longitudinal. The enrolled subjects were divided into the following categories: healthy (H) (all individuals who were not osteoporotic, not sarcopenic, not osteosarcopenic), osteoporotic (OP) (all individuals with a T-score < −1 SD), sarcopenic (SP) (all individuals who fall within the various definitions of sarcopenia), and osteo-sarcopenic (OSP) (all individuals with osteopenia/osteoporosis and sarcopenia simultaneously). The group of uncategorised (RG) was also considered, i.e., patients who had no direct or clear allocation in the primary study to one of the four previous groups.

### 3.3. Analysis Outcome

**Figure 2:**
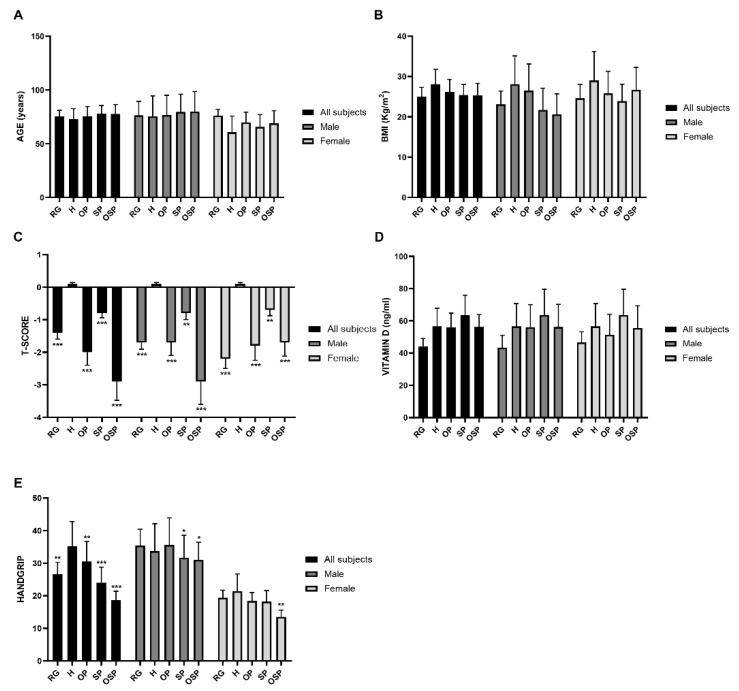
Distribution of the variables associated with the subjects of the primary studies. (**A**) Age variable; (**B**) BMI variable; (**C**) T-score variable; (**D**) Vitamin D; (**E**) Handgrip variable. All subjects and male and female groups were analysed separately. Differences were considered significant when the p value was < 0.05 (* *p* <0.05, ** *p* <0.01, *** *p* <0.001).

**Figure 3:**
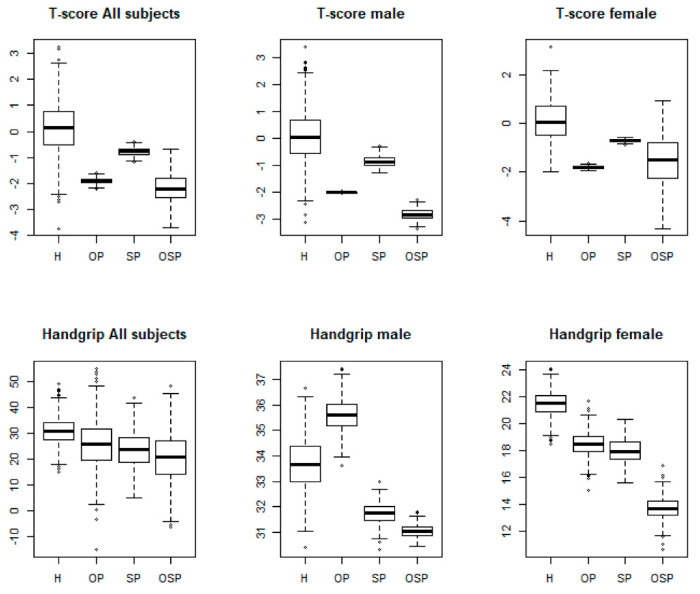
Boxplot of the statistically significant correlation between the T-score and handgrip variables in all subjects, males, and females between groups (χ^2^ = 188; ** *p* < 0.001) and within the single OSP group (χ^2^ =2 78; ** *p* < 0.001).

Analyses of the mean values between groups (all subjects, males, and females) showed important differences with respect to age and clinical covariates (Figure 2). Regarding the variables age (panel A), BMI (panel B), and vitamin D (panel D), the analysis showed no significant differences between the five different categories of subjects, nor between males and females. In contrast, we found significant differences for the variable T-score (panel C) and handgrip (panel E) between all groups. We observed a progressively decreasing trend for the T-score values, starting from the H group towards the OSP group, in all subjects (H: 0.1 ± 0.04; OP: −2.0 ± 0.4; SP: −0.8 ± 0.14; OSP: −2.9 ± 0.6) (Table 2). We also obtained the same result by analysing the T-score value separately between males and females (male: H: 0.1 ± 0.04, OP: −1.7 ± 0.4, SP: −0.8 ± 0.2, OSP: −2.9 ± 0.7; female: H: 0.1 ± 0.04, OP: −1.8 ± 0.45, SP: −0.7 ± 0.18, OSP: −1.7 ± 0.42). In relation to the RG group, we found the following T-score values: all subjects, −1.4 ± 0.2; male, −1.7 ± 0.21; female, −2.2 ± 0.3. The same decreasing trend was also observed for the variable handgrip, with significant differences between the different groups: in all subjects: H: 35.2 ± 7.6, OP: 30.5 ± 6.13, SP: 24.0 ± 4.8, OSP: 18.7 ± 2.7; in the male group: H: 33.7 ± 8.4, OP: 35.6 ± 8.3, SP: 31.7 ± 6.9, OSP: 31.0 ± 5.4; in the female group: H: 21.4 ± 5.3, OP: 18.4 ± 2.6, SP: 18.2 ± 3.4, OSP: 13.5 ± 2.1. In relation to the RG group, we found the following handgrip values: all subjects, 26.6 ± 3.7; male, 35.4 ± 5.0; female, 19.4 ± 2.3. After analysing the distribution of the variables considered in this meta-analysis, we determined an integrated database representing a sample of the population under analysis, according to the clusters defined a priori (H, OP, SP, and OSP) and the data-pooling conducted. The purpose of the classification adopted responds to the need to use an elected sample according to the criteria highlighted above to initiate association analyses between the variables under study. This sample was constituted by taking the outcome of the individual primary studies for the selected variables and by risk group where present and inserting the weighted or global outcome for the missing cases. The sample was then reclassified using cut-offs that meet the EWGSOP2 and AWGS criteria for sarcopenia and the criteria established by the WHO for osteoporosis [14,15]. The modalities of the qualitative variables are represented by the outcome estimated from the 16 individual primary studies. From an initial analysis of the sample, a statistically significant correlation was observed between two of the analysed variables, T-score, and handgrip among all groups (rho = 0.47; *p* < 0.001). Furthermore, this correlation was also found within the individual OSP group (rho = 0.17; *p* < 0.001). Finally, the association between these variables was analysed by means of the chi-squared independence test, applied to the contingency tables obtained through the classification adopted previously. The results of this statistical analysis confirmed the data obtained previously, showing the existence of a significant association between the two variables, T-score and handgrip, both between groups (χ^2^ = 188; *p* < 0.001) and within the single OSP group (χ^2^ = 278; *p* < 0.001). The boxplots show the distribution of the two variables, by gender and risk group, obtained from the sample analysed (Figure 3).

## 4. Discussion

### 4.1. Summary of Evidence

Osteosarcopenia is a newly defined pathology in which two of the most characteristic diseases of the elderly population, osteoporosis and sarcopenia, coexist [5]. Osteoporosis is characterised by a reduction in bone mineral density (BMD) and a deterioration of the microarchitecture of the bone tissue, while sarcopenia is manifested by a loss of muscle mass, strength, and function (19). While there are already valid criteria on which to base a rapid and accurate diagnosis of osteoporosis and sarcopenia, there is no uniform protocol for diagnosing osteosarcopenia, this being a multifactorial and, as mentioned above, recently defined condition. The aim of this meta-analysis was to identify a correlation between the variables that are usually considered to establish a diagnosis of osteoporosis (T-score and vitamin D) and the result of the main diagnostic test that is carried out to establish a possible condition of sarcopenia (handgrip strength test). The analysis of variables’ trend between the categories of subjects taken into consideration showed significant differences between the different groups for two of the five variables investigated, T-score, and handgrip strength test. This result highlights how the trend of these variables goes hand in hand with the progressive increase in the severity of the osteoporotic and sarcopenic condition, up to osteosarcopenia. Next, the reclassification of the sample through the global size effect confirmed the hypotheses of causal association between the selected groups and the variables, as well as between the variables themselves within the groups. These results enrich the matrix of the meta-analysis by providing different information. In fact, through the calculation of the global effect sizes of the variables, new threshold values were identified, against which different levels of risk attributable to each group defined a priori are associated. These results were tested by reconstructing a sample of individuals and assessing the association between the different variables and between risk groups. Some studies found associations only between some single variables such as BMD [31], chair stand test [23], and handgrip strength [30]. Instead, our meta-analysis showed a very significant association between handgrip strength and T-score (*p* < 0.001), as well as their mutual influence. This result suggests that poor bone quality, as evidenced by negative T-score values, as well as reduced muscle strength and function, as evidenced by poor handgrip strength test results, may concomitantly lead to osteosarcopenic syndrome. This correlation could therefore represent a potential new parameter that the clinician could use to make a diagnosis of osteosarcopenia, quickly and inexpensively.

### 4.2. Limitations of This Review

Since osteosarcopenia is a newly defined syndrome, few studies are currently available in the literature. This aspect therefore represents the main limitation of this meta-analysis, in addition to other limitations. Firstly, the analysed studies do not all belong to the same type. Furthermore, there are both prospective and retrospective studies but also longitudinal, cross-sectional, and observational studies and no randomised studies. The population is heterogeneous, comprising healthy volunteers, non-hospitalised or institutionalised (community-dwelling individuals); fracture patients; patients with a history of falls; and hospitalised patients. Additionally, their ethnicity, country or region of origin, risk factors, and treatment were not considered. The purpose of the classification through the global ES responds to the need to use an elected sample according to the considered criteria in order to conduct an association analyses between the variables under study. However, a much larger sample size will be required to confirm the type and level of association measured through the statistics used in this review. In the data collection, different criteria were used in defining sarcopenia, although looking at the cut-offs of the variables under consideration, we considered it legitimate to accept the various groups of sarcopenic patients in each study as such. Moreover, further primary studies are needed to increase the sample size by filling the gaps in the availability of data for specific variables to assess the intensity of this interesting relationship.

### 4.3. Implications for Clinical Practice

**Figure 4:**
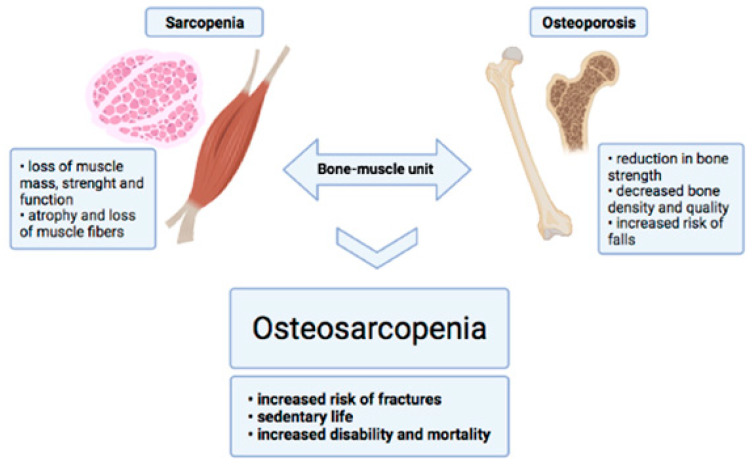
Bone–muscle unit. Characteristics of sarcopenia and osteoporosis diseases, which contribute to the onset of osteosarcopenia (created with BioRender.com).

As mentioned above, osteosarcopenia is a recently defined condition for which no protocol has yet been established to allow for a rapid diagnosis. This pathology affects both bone and muscle tissue, which together make up the bone–muscle unit (Figure 4). An impairment in the quality of muscle tissue inevitably leads to degeneration of bone tissue, and vice versa. The results of this study further highlight how bone–muscle crosstalk is implicated in the pathogenesis of osteosarcopenia, suggesting a new diagnostic strategy in which bone and muscle tissue assessment parameters converge. Thus, this study provides a new potential tool that could speed up the elaboration of a diagnosis of osteosarcopenia, immediately highlighting which aspects need to be addressed with therapeutic treatment [34].

### 4.4. Implications for Further Research

It is suggested that future research should be strengthened with the help of these two variables in the context of risk factors associated with osteosarcopenia. For this purpose, multivariate regression models, including logistic models, will be required. Through such models, regression coefficients associated with the variables and their modes will be available: the value and the sign will provide information about the strength and direction of their influence on the risk factors. The final hope is to acquire the capacity to elaborate a predictive, single model, increasingly used in the medical and health field, in order to respond specifically to the needs of the individual, implementing all the tools that can make this possible.

## 5. Conclusions

Our pilot study identified a new potential predictive model, on the basis of the correlation of the two variables T-score and handgrip strength, for a faster and more targeted diagnosis of osteosarcopenia, increasing the spectrum of clinical investigation.

## Figures and Tables

**Table 2:** Characteristics of the different subgroups of the studies.

	RG	H	OP	SP	OSP
All subjects	Mean ± SD	Mean ± SD	Mean ± SD	Mean ± SD	Mean ± SD
AGE	75.5 ± 5.5	73.0 ± 9.6	75.6 ± 8.9	78.0 ± 7.5	77.6 ± 8.8
BMI	25.0 ± 2.3	28.1 ± 3.7	26.2 ± 3.1	25.4 ± 2.6	25.3 ± 3.0
T-SCORE	−1.4 ± 0.2	0.1 ± 0.04	−2.0 ± 0.4	−0.8 ± 0.14	−2.9 ± 0.58
VITAMIN D	43.9 ± 5.1	56.6 ± 11.2	56.0 ± 8.8	63.6 ± 12.3	56.2 ± 7.6
HANDGRIP	26.6 ± 3.7	35.2 ± 7.6	30.5 ± 6.13	24.0 ± 4.8	18.7 ± 2.7
Male	Mean ± SD	Mean ± SD	Mean ± SD	Mean ± SD	Mean ± SD
AGE	76.3 ± 13.0	75.5 ± 18.9	76.6 ± 18.4	79.5 ± 16.5	79.8 ± 18.7
BMI	23.1 ± 3.3	28.1 ± 7.0	26.5 ± 6.6	21.7 ± 5.4	20.6 ± 5.1
T-SCORE	−1.7 ± 0.21	0.1 ± 0.04	−1.7 ± 0.4	−0.8 ± 0.2	−2.9 ± 0.7
VITAMIN D	43.4 ± 7.6	56.6 ± 14.1	56.0 ± 14.0	63.6 ± 15.9	56.2 ± 14.0
HANDGRIP	35.4 ± 5.0	33.7 ± 8.4	35.6 ± 8.3	31.7 ± 6.9	31.0 ± 5.4
Female	Mean ± SD	Mean ± SD	Mean ± SD	Mean ± SD	Mean ± SD
AGE	76.2 ± 5.8	60.6 ± 15.1	69.8 ± 9.5	65.6 ± 11.4	68.9 ± 11.8
BMI	24.6 ± 3.4	29.0 ± 7.2	25.8 ± 5.5	23.9 ± 4.2	26.7 ± 5.6
T-SCORE	−2.2 ± 0.3	0.1 ± 0.04	−1.8 ± 0.45	−0.7 ± 0.18	−1.7 ± 0.42
VITAMIN D	46.6 ± 6.6	56.6 ± 14.1	51.3 ± 12.8	63.6 ±15.9	55.5 ± 13.8
HANDGRIP	19.4 ± 2.3	21.4 ± 5.3	18.4 ± 2.6	18.2 ± 3.4	13.5 ± 2.1

## Data Availability

The data presented in this study are available on request from the corresponding author.
